# Exploring New Patient Understanding of Osteopathic Manipulative Medicine using a Cross-Sectional Survey and Mixed Methods Approach

**DOI:** 10.51894/001c.37924

**Published:** 2022-09-06

**Authors:** Jared Ham-Ying, Samuel J Wisniewski, Jake Rowan, Izabela Birsanescu, Alicia Speak, Rebecca Malouin

**Affiliations:** 1 Osteopathic Principles and Practices California Health Sciences University; 2 Statewide Campus System Michigan State University; 3 Osteopathic Medicine Michigan State University; 4 College of Osteopathic Medicine Michigan State University; 5 College of Human Medicine Michigan State University

**Keywords:** Osteopathic medicine, Osteopathic manipulative treatment, Professional identity, Osteopathic practice, Osteopathic survey, Osteopathic perception nurses, Osteopathic literature review, manual healing, manipulation, Osteopathy, hands-on medicine, OMM, ONMM, OMT, osteopathic neuromusculoskeletal medicine, neuromusculoskeletal medicine

## Abstract

**INTRODUCTION:**

Osteopathic manipulative medicine (OMM), a patient-focused approach to medical treatment utilized by doctors of osteopathic medicine (DO), continues to be an under-utilized resource in treating musculoskeletal disorders. Lack of familiarity by both referring physicians and patients of OMM can impact patient-physician communication and impede patient-centered care approaches. This 2020-2021 study was conducted to investigate new patient understanding of OMM within the Michigan State University OMM Clinic.

**METHODS:**

A set of 18 survey questions developed by the authors within their MSU OMM clinic were utilized for the study. The main purpose of the questions was to generally ascertain new patient’s understanding of OMM, its role in patient care, who can place clinic referrals and the services an OMM clinic provides. Respondents were new adult patients at the MSU OMM clinic.

**RESULTS:**

The convenience study sample of 83 respondents was primarily female, 60 (72.3%). Few respondents were familiar with OMM, as only eight (9.64%) reported prior experience with OMM. Of the 83 patients in this study, 69 (80.7%) reported back pain. When examining referral patterns, there were low numbers of referrals from non-PCP providers, especially from advanced practice providers (APP), including physician assistants (n = three, 3.66%) and nurse practitioners (n = eight, 7.96%). Most surveyed patients 61 (73.5%) had been referred by their primary care providers.

**CONCLUSIONS:**

Results indicate that communication directed to non-DO physicians, advanced practice providers and patients about OMM services may be helpful to provide noninvasive symptomatic treatment options for musculoskeletal conditions. Further larger-scale studies examining both non-DO provider and new patient perceptions concerning OMM are clearly warranted.

## INTRODUCTION

Medical licensure in the United States is currently obtained by both Doctors of Osteopathic Medicine (DO) and Medical Doctors (MD) i.e., “allopath’s”. In addition to base licensure requirements, DO physicians complete formal training in Osteopathic Manipulative Medicine (OMM) as part of their medical education.^1^ OMM is a patient-focused approach to medical treatment that includes the application of an osteopathic philosophy, structural diagnosis, and set of hands-on techniques known as osteopathic manipulative treatment (OMT) to diagnose, treat, and prevent disease.^1^

After the civil war, Andrew Taylor Still, MD, founded osteopathy based on the belief that the body has an innate ability to heal itself and that the musculoskeletal system plays an integral part in a person’s health.^2^ Still’s philosophy maintains that the body is more than the sum of its parts and that doctors need to focus on caring for the whole patient, rather than the disease alone.^1^

Neuromuscular conditions including headaches, neck, back, shoulder, and/or other joint pain, present as a significant disease burden for Americans. According to the 2012 National Health Interview Survey (NHIS), 125 million adults, more than half of Americans, suffer from musculoskeletal conditions.^3^ According to the CDC’s occupational health data in 2015, the prevalence of low back pain is 14% in working adults.^4^ Although OMT has been shown in numerous studies to be an efficacious treatment modality for musculoskeletal disorders (e.g., neck, shoulder and back pain) and other neuromuscular complaints (e.g., headaches), it continues to be an underutilized medical care resource.^5–10^

In a 2016 National Health Interview Study, although 54% of adults reported a musculoskeletal complaint, only 18% reported using a practitioner-based treatment option, such as osteopathic or chiropractic manipulation.^3^ Currently about 11% of all U.S. physicians are DO’s,^1^ with an estimated number of practicing DO’s in 2021 to be nearly 135,000 (i.e., an 80% increase in the past decade).^11^ According to the 2021 AOA Physician Masterfile, an estimated 7,445 osteopathic medical students graduated in 2021.^11^

Lack of familiarity by both referring physicians and patients of OMM can impact both patient-physician communication and impede patient-centered care approaches. In addition, earlier studies have demonstrated that those with a more comprehensive accurate understanding of OMM may be more likely to seek OMT services.^12,13^

### Study Purpose

This study was conducted to investigate new patient understandings of OMM within the Michigan State University OMM Clinic (MSU OMM). The results of this study would ideally be used to improve patient education about OMM and tailor communication to help potential referring physicians and patients better understand the potential role of DO’s and OMM in their medical care.

## METHODS

A set of 18 survey-type questions was developed by the study authors in collaboration with attending physicians and resident physicians within the MSU OMM clinic. Many survey items were modified from prior OSTEOSURV studies conducted by the Licciardone group.^14–16^ The majority of study data reported in this paper were extracted by authors JH and IB from the clinic’s new patient intake form with several additional open-ended questions added at the end of the provided consent form.

The questions taken from the intake form (Appendix A) included questions concerning who referred the patient, why they were referred, what physical complaint(s) they were being seen for, as well as their prior treatments and treatment expectations. Several non-identifiable demographic questions were included. (see Appendix A for full survey instrument)

The new patient intake, authorization, and consent forms were either mailed or emailed to new patients, depending on patient preference, by clinic staff. Patients were consented at time of initial appointment, by the participating clinic physicians involved in this study (authors JH and JR). The forms and clinical treatment data were collected by authors JH and JR and placed in a secure key locked storage filing cabinet located in the clinic but separate from the patient population.

Respondents were all new adult patients of the MSU OMM clinic in East Lansing, Michigan. Before data collection, the authors had received IRB approval for the study design. All respondent data was de-identified by authors JH and IB. Patient recruitment and data collection took place between October 2020 and March 2021.

### Data Analytics

**Quantitative Data:** Base descriptive statistics, i.e., rates and frequencies were provided for the demographic characteristics of age and gender. In addition, descriptive statistics were also calculated for education level, whether the respondent had any chronic medical issues, whether they had any previous experience with visiting an OMM clinic, chief somatic complaints, average (mean) daily pain ratings, patient defined treatment success, as well as information regarding their referring physicians’ specialty and licensure type. Author SJW performed all quantitative data analytics using IBM SPSS Software Version 26.

**Qualitative Data:** A framework methodology was utilized for qualitatively exploring the data, specifically centered around the survey question of, “What were you told by your referring physicians about this clinic and the services provided here?”. Authors JH, SJW, and JR completed the qualitative analyses of response data using a framework analysis approach, (i.e., an approach consisting of familiarization/identifying a thematic framework, indexing, charting, and mapping & interpretation).^17^

Each author first separately reviewed the patient open-ended question response data, identifying initial coding “themes” present in the response. Next, the three authors met as a group to compare and discuss their initial theme choices and refine the thematic framework/indexing/coding list.

## RESULTS

The total convenience sample size prior to patient exclusions was 192. From this total of 192, 10 (5.2%) patients were excluded from the study due to having incomplete paperwork and 99 (51.5%) were excluded due to missing pages from their paperwork. A final total of 83 (43.0%) of eligible patients were included in this study. The estimated total number of new patients seen each month at the authors’ clinic during the study period was estimated to be 340.

Respondents’ average age was 45.2 (SD = 15.7), with a tertile breakdown of: 26 (31.3%) between the ages of 18 to 35, 29 (34.9%) between 36 to 51 years, and 28 (33.7%) between 52 to 79 years old. A subgroup of 60 (72.3%) respondents were female, 22 (26.5%) were male, and one (1.2%) person reported an “Other” response option. Education levels were self-reported as; “GED/high school degree,” eight (9.6%), “some college,” 18 (21.7%), “college degree,” 29 (34.9%), and “postgraduate,” 23 (27.7%). No information on education level was provided from five (6.0%) participants.

When asked about their chronic medical issues, 45 (54.2%) of respondents indicated that they had been diagnosed with one or more chronic health issues, while 31 (37.3%) denied possessing any chronic health issues. Most survey respondents (i.e., n = 74, 89.2%), reported that they did not have previous experience visiting an OMM office. (Table 1)

**Table 1.** Participant Demographic Characteristics

	**N = 83**
**Age (mean)**	45.2 (SD 15.7)
*Range*	18 – 79
**Gender**	
*Male*	22 (26.5%)
*Female*	60 (72.3%)
*Other*	1 (1.2%)
**Education**	
*GED/High School Degree*	8 (9.6%)
*Some college*	18 (21.7%)
*College degree*	29 (34.9%)
*Postgraduate*	23 (27.7%)
**Chronic Medical Problems**	
*Yes*	45 (54.2%)
*No*	31 (37.3%)
*Did not answer*	7 (8.4%)
**Prior OMM Experience?**	
*Yes*	8 (9.64%)
*No*	74 (89.2%)
*Did not answer*	1 (1.20%)

SD = standard deviation

When asked about their primary complaint (i.e., “chief” complaint) that had brought them to the OMM offices, 35 (41.7%), respondents reported that they had two or more complaints that included back pain (i.e., either upper or lower). For upper back pain alone, 16 (19.3%) patients reported this as their primary reason, or “chief complaint”, for visiting the OMM office. Similarly, 16 (19.3%) also choose lower back pain as their sole chief complaint. Finally, both “Head pain” and “Other” were listed by eight (9.6%) respondents as the primary reason for visiting the OMM office. (Table 2)

**Table 2.** Sample Patient Chief Complaints

**Chief Complaint**	**N (%)**
*Upper Back Pain*	16 (19.3%)
*Lower Back Pain*	16 (19.3%)
Two + complaints including back pain*	35 (41.7%)
*Head Pain*	8 (9.6%)
*Other*	8 (9.6%)
*Total*	83

*back pain refers to either upper back pain, lower back pain, or both

When asked to rate their average daily pain on a 0 to 10 scale (0 being “no pain” and 10 being “the worst pain”), respondents’ average pain was 4.7 (SD = 2.2), with a range of reported scores between zero to 10. Eighteen (21.7%), respondents reported a pain level of “3”, followed closely by a pain level of “5”, with 15 (18.1%) patients self-reporting a pain level in the overall middle of the scale.

Using a question directly from the standard “new patient intake form”, respondents defined their treatment “success” as follows: “Freedom from all pain” (n = 37, 44.6%), “Doing all desired activities” (n = 34, 41.0%), “Any amount of pain relief” (n = 36, 43.4%), and “Tolerating simple activities”, (n = 18, 21.7%) . It should be noted by readers that patients could (and often did) select more than one option.

When asked about their referring physician, most patients (n = 61, 73.5%) reported that they had been referred by their primary care provider. The remaining referring physicians were from physical medicine and rehabilitation, seven (8.4%), surgical specialties, six (7.2%), neurology, six (7.2%), and rheumatology, three (3.6%). (Table 3)

**Table 3.** Referring Provider Practice Areas

**Referring Provider Practice Specialty Area**	**N (%)**
*Primary Care Provider*	61 (73.5%)
*Physical Medicine*	7 (8.4%)
*Neurology*	6 (7.2%)
*Surgical Specialties*	6 (7.2%)
*Rheumatology*	3 (3.6%)
*Total*	83

Patients were also asked about the specific licensure type of their referring physicians/providers. The majority had been referred by DO’s (n = 43, 52.4%), followed by M.D.’s, (n = 28, 34.1%). (Table 4)

**Table 4.** Referring Provider Licensure Types

**Licensure**	**N (%)**
*DO*	43 (52.4%)
*MD*	28 (34.1%)
*NP*	8 (9.76%)
*PA*	3 (3.66%)

DO = Doctor of Osteopathic Medicine

MD = Medical Doctor

NP = Nurse Practitioner

PA = Physician’s Assistant

### Qualitative Analyses

#### Initial Indexing/Coding

Authors JH, SJW, and JR each reviewed the individual responses to the survey question, “What were you told by your referring physician about this clinic and the services provided here?” A total of 69 (83.1%) respondents provided a response to this open-ended question. Each author first separately reviewed the patient open-ended question response data, identifying initial coding “themes” present in the response.

Next, the three authors met as a group to compare and discuss their initial theme choices and refine the thematic framework/indexing/coding list.

Although an initial list of ten terms was compiled, this list was refined by group consensus to combine “generic term OMM” with “Partial ONMM description” and separating the one mention of “Manipulation/Alignment” to count simply towards, “Manipulation”, leaving a total of eight indexing/coding terms. (Table 5)

**Table 5.** Qualitative Framework Analyses: Frequency Summary of coding terms used by new OMM patients

**Index/Code**	**Total**
*Alignment*	6
*Alternative treatment*	3
*Chiropractic*	11
*Generic term OMM/Partial ONMM description*	11
*Indirect recommendation*	5
*Manipulation*	13
*Patient asked for referral*	5
*Symptom relief*	35

OMM = Osteopathic Manipulative Medicine

Note: Total of 89 terms as some patients provided more than one “code”.

In general, female respondents (n = 10, 19.6%) tended to be more familiar with general OMM terminology and or OMM descriptors than male respondents, (n = one, 4.6%). In addition, female patients (n = five, 9.8%) apparently more often either directly asked for the OMM referral (i.e., zero direct requests from male patients), or indirectly female (n = five, 9.8%) vs. zero for males. (Table 6)

**Table 6.** Frequency of OMM Referral Codes by Gender

**Index/code**	**Male** **(N = 19)**	**Female** **(N = 51)**	**Total**
*Alignment*	2 (10.5%)	4 (7.8%)	6
*Alternative treatment*	-	3 (5.9%)	3
*Chiropractic*	2 (10.5%)	9 (17.6%)	11
*Generic term OMM/Partial ONMM description*	1 (4.6%)	10 (19.6%)	11
*Indirect recommendation*	-	5 (9.8%)	5
*Manipulation*	4 (21.1%)	8 (15.7%)	13
*Patient asked for referral*	-	5 (9.8%)	5
*Symptom relief*	11 (57.9%)	24 (47.1%)	35

*One additional respondent, who self-identified as “AFAB/genderqueer” responded under what was coded “Manipulation”

OMM = Osteopathic Manipulative Medicine

Finally, these eight codes were examined by age tertile, i.e., the age distribution of the patient sample divided into three (roughly) equal groups. (Table 7)

**Table 7.** Frequency of Codes by Age Group

**Index/code**	**18 – 35** **Years**	**36 – 51** **Years**	**52 – 79** **Years**	**Total**
*Alignment*	3 (9.09%)	2 (6.45%)	1 (4.00%)	6
*Alternative treatment*	1 (3.03%)	1 (3.23%)	1 (4.00%)	3
*Chiropractic*	6 (18.2%)	4 (12.9%)	1 (4.00%)	11
*Generic term OMM/Partial ONMM description*	3 (9.09%)	5 (16.1%)	3 (12.0%)	11
*Indirect recommendation*	-	1 (3.23%)	4 (16.0%)	5
*Manipulation*	4 (12.1%)	4 (12.9%)	5 (20.0%)	13
*Patient asked for referral*	2 (6.06%)	2 (6.45%)	1 (4.00%)	5
*Symptom relief*	14 (42.4%)	12 (38.7%)	9 (36.0%)	35

*Note: Some patients provided more than one “code”

### Qualitative Framework Analyses: Charting

Next, authors JH, SJW, and JR developed “charting” categories. The eight index “codes” from Table 7 above were then categorized under four themes: “Method of referral”, which included both indirect recommendations and patient requested referrals, 10 (12.05%), “Specialty-related terminology”, which included alignment, manipulation, and generic term OMM/partial ONMM descriptors, 31 (37.3%), “Adjacent therapy familiarity”, chiropractic and alternative treatment, 14 (1.9%), and “Addressing patient complaints”, which included symptom relief, 35 (42.2%).

### Qualitative Framework Analyses: Referral Mapping

For the survey item, “What were you told by your referring physicians about this clinic and the services provided here?”, 69 (83.1%) of respondents provided an answer to this question. Broadly speaking, the most frequently cited theme was from 35/69 (50.7%) of patients who were told that OMM could in some way contribute to mitigating their symptoms. Of the 10/69 (14.5%) who specifically mentioned how a referral was initiated, there was an equal split of five patients who requested the OMM referral themselves, and five who reported that their OMM clinic referral had come from an “indirect recommendation.” All ten patients who requested the OMM referral themselves were female.

## DISCUSSION

This study was designed to better understand patient perception of OMM, as well as inform future efforts in patient and provider outreach regarding the utility of OMT. Sample respondents reported having been largely unfamiliar with OMM, as only eight (9.64%) reported prior experience with OMM. The fact that those in the sample had already been referred to the authors’ OMM clinic may reflect the general publics’ limited awareness and understanding of OMM.

As demonstrated in numerous prior US studies, musculoskeletal pain, particularly back pain, is a major concern for adults.^3,4^ Of our 83 study patients, 69 (80.7%) respondents reported some form of back pain, with the other 16 (19.3%) patients complaining of different kinds of musculoskeletal and neuromuscular pain. Patients reported an average pain level of 4.7 out of 10, apparently high enough for them to seek care and receive a provider referral to the MSU OMM clinic.

Earlier studies have demonstrated how OMM services remain vastly underutilized even though such services have been shown to effectively decrease pain, need for pain medication and improve patient comfort/recovery.^11,18–20^ For example, one smaller sample 2019 pilot study has shown how patient willingness to receive OMM services can be increased after reading a brief knowledge-based brochure.^21^

When examining referral patterns, we found relatively low proportionate numbers of referrals from non-PCP providers, especially from advanced practice providers. We found more respondents i.e., (61 of 83, or 73.5%) were referred by their primary care provider and/or DO physicians. Prior studies have already shown lower overall opinions of DO physicians among patients and healthcare professionals when compared to other MDs, physician assistants and nurse practitioners.^22,23^ Future cross-discipline educational initiatives or programs regarding osteopathic medicine may therefore prove key to increase utilization of OMM.

Interestingly, only 10 (14.5%) patients asked for a referral to OMT, either by directly asking, or by referral to the clinic by a family member or friend, all of whom were women. While this may be due to the female skew (i.e., 72.3%) of our survey sample, this low number of requests for treatment also highlights the importance of increasing public awareness of osteopathic medicine and OMM through enhanced education and outreach from the osteopathic profession.

### Study Limitations

Readers’ interpretation of our findings may be limited by several factors. First, this study was conducted during a 2020-2021 high COVID-19 outbreak period in Michigan.^24^ Of those participants initially surveyed, an excluded subset of 109 (57.0%) of initial respondents were excluded by the authors due to missing/incomplete paperwork. Both issues could perhaps be more fully addressed in the future with electronic data collection to reduce numbers of direct patient contacts and ensure more complete provider documentation. As already indicated, our sample was predominately female, so it is unclear whether our findings may be generalizable to more heterogenous clinic populations. Finally, data was unavailable for frequencies of the number of patients contacted who responded to either letters or emails. This lack of data regarding response rates for method of follow up limits the authors’ ability to present whether those being contacted via postal mail (i.e., letter) or email were more or less likely to respond to this survey request.

## CONCLUSIONS

Based on these results, more direct and targeted, communications to non-DO physicians, advanced practice providers and patients about benefits of osteopathic medicine as a noninvasive treatment option for musculoskeletal conditions are indicated. Future larger sale studies with varied patient and provider samples to systematically examine factors influencing perceptions of OMM are warranted.

### Conflict Of Interest

None.

## Appendix A

Survey Instrument

1. What is your level of education?
  - pre/some high school
  - high school/GED
  - some college
  - college degree
  - post-graduate
2. What is your age?
  - 0-18
  - 19-65
  - Over 65
3. What is your gender?
  - Male
  - Female
  - Other
4. What type of insurance do you have?
5. Who was your referring physician and what is their specialty?
6. What were you told by your referring physician about this clinic and the services provided here?
7. What is your understanding of why you were referred to this clinic?
8. What do you expect to gain/achieve from this visit?
9. Please list which other professions you have seen prior to us for this issue?
10. What are you being seen for at this clinic today?
  - **State the injury type, pain site, symptoms or circle don’t know**
